# Antitrypanosomal activity of *Verbascum sinaiticum* Benth. (Scrophulariaceae) against *Trypanosoma congolense* isolates

**DOI:** 10.1186/s12906-016-1346-z

**Published:** 2016-09-15

**Authors:** Ermias Mergia, Workineh Shibeshi, Getachew Terefe, Tilahun Teklehaymanot

**Affiliations:** 1 Department of Pharmacology and Clinical Pharmacy, Faculty of Health Sciences, Addis Ababa University, Addis Ababa, Ethiopia; 2 Department of Parasitology, Faculty of Veterinary Medicine, Addis Ababa University, Addis Ababa, Ethiopia; 3 Aklilu Lemma Institute of Pathobiology, Addis Ababa University, Addis Ababa, Ethiopia

**Keywords:** *Verbascum sinaiticum*, *Trypanosoma congolense*, In vivo, Phytochemical, Ethiopia

## Abstract

**Background:**

African Trypanosomiasis is a neglected tropical disease with a large impact on the livelihood of the rural poor in Sub-Saharan Africa. The available drugs for managing this disease are old, expensive and are facing the problem of drug resistance. Thus, the aim of this study was to evaluate in vivo antitrypanosomal efficacy of aqueous and absolute methanol leaf extracts of *Verbascum sinaiticum* Benth. against *Trypanosoma congolense* field isolate.

**Methods:**

*Verbascum sinaiticum* (Local name ‘*qetetin*a’) is a biennial plant, and 60–150 cm tall. It is traditionally used to treat wound, stomachache, viral infection, cancer, sunstroke, fever, abdominal colic, diarrhea, hemorrhage, anthrax, and hepatitis. The efficacy of aqueous and absolute methanol leaf extracts of *V. sinaiticum* was evaluated in a randomized experiment with Swiss albino mice infected with *T. congolense* field isolate. The extracts were administered at doses of 100, 200 and 400 mg/kg by intraperitoneal injection for seven days at 12 Days Post-Infection (DPI) when the peak parasitaemia level was approximately 10^8^ trypanosomes/ml. Parasitaemia, Packed Cell Volume (PCV), mean survival time and change in body weight were used as indices for monitoring the efficacy of the extracts. Diminazene (28 mg/kg) was used as a positive control while 2 % Tween was used as the negative control. Phytochemicals screening were conducted following standard methods.

**Results:**

The extracts showed no toxicity effect in Swiss albino mice and had LD_50_ above 2000 mg/kg. The phytochemicals screened in *V. sinaiticum* were alkaloids, flavonoids, glycoside, saponins, steroids, phenolic compounds, and tannins. The mice treated with absolute methanol leaf extract of *V. sinaiticum* at 400 mg/kg dose had significantly lower mean parasitaemia (7.20 ± 0.16) (*p* < 0.001) as compared to the negative control group (8.82 ± 0.12) on day 14 of treatment. Animals treated with the same dose had significant (*p* < 0.001) higher PCV value and body weight and as well as the highest mean survival time of 40.20 ± 0.31 days as compared to the negative control at the end of the observation period.

**Conclusion:**

This study established that *Verbascum sinaiticum* had trypanocidal activity. The crude extracts have partially eliminated trypanosomes in a dose-dependent manner. The study can be a basis for future in-depth analysis of the biologically active chemicals.

## Background

African Animal Trypanosomiasis (AAT) is a neglected tropical disease caused by *Trypanosoma brucei brucei, T. vivax,* and *T. congolense* while human Trypanosomiasis is caused by two subspecies of *Trypanosoma brucei: T. brucei gambiense,* and *T. brucei rhodesiense* [1, 2]. The disease has an enormous economic impact in Africa since it affects the settlement patterns of people including land use and farming [3, 4]. In Ethiopia, AAT is one of the most significant and costly diseases because it hinders the effort made by the government to attain food sufficiency and affect the greater river basins of Abay, Omo, Ghibe, and Baro that have a high potential for agricultural development [5–8].

Trypanocides are used for the control of the disease in 37 African countries where animal trypanosomiasis is endemic but the available drugs are old, expensive, less effective, and face the problem of drug resistance [9–11]. The continued use of the same Trypanocides for years has resulted in drug resistance that has been largely responsible for the current chemotherapeutic failures in Ethiopia [9, 12]. Therefore, there is a need to develop alternative and efficacious drugs, either synthetically or from plant origins.

Herbal remedies are known to have been used for the treatment of this disease such as. *Khaya senegalensis, Piliostigma reticulatum*, *Securidaca longepedunculata, Ximenia americana* and *Artemisia abyssinica* [2, 13, 14] and are strongly trypanocidal to *T. b. brucei* and *T. congolense*. In Ethiopia, recent reports indicated that some medicinal plants have antitrypanosomal activity and the potentials of many of these plants are yet to be investigated and claims to be tested [15–19]. Thus, this study was designed to determine the in vivo antitrypanosomal activity of *V. sinaiticum* against *T. congolense* infected mice. This study will also seek to validate results from the previous study by Mergia et al. [20] that gave indications of in vitro antitrypanosomal activity of methanol crude leaf extracts of *V. sinaiticum* against *T. congolense* field isolate.

*Verbascum sinaiticum* is traditionally used as a treatment for wound, stomachache [21]; viral infection, cancer [22]; sunstroke fever, abdominal colic, diarrhea, hemorrhage, anthrax [23]; hepatitis; and as hepatoprotective [24, 25]. Moreover, powder leaf of *V. sinaiticum* mixed with water is given orally [22, 26] and the filtrate is added to left ear and nose of animals [23] for the treatment of animal trypanosomiasis. The species in genus *Verbascum* are well-known drugs in folk medicines.

## Methods

### Reference drug and chemicals

The commercial trypanocidal drug used was diminazine aceturate (*Veriben®* containing 1.05 g diminazine aceturate *+* 2.36 g antipyrine, (Ceva Santé Animale, France; batch number- 719A1).

## Plant material

### Collection of plant specimen

Voucher specimens and fresh leaves of *V. sinaiticum* were collected, in March 2013, from Entoto-mountain about 10 km north of Addis Ababa, Ethiopia. The taxonomic identification was done by an expert using Taxonomic keys and herbaria samples and deposited (Collection EM/002) at the National Herbarium of Addis Ababa University. The fresh leaf samples were prepared by air drying, grinding, and kept in plastic bags until required.

### Preparation and storage of extracts

The aqueous and absolute methanol extracts were prepared by macerating 200 g of dried leaf powder of *V. sinaiticum* with 1000 ml of each solvent for 48 h. After filtering using Whattman No. 3 filter paper (Whattman Ltd., England), the aqueous extract was placed on a Petri dish and lyophilized for seven days to yield a solid residue. The absolute methanol extract was dried using Rotavapor (BÜCHI Rotavapor, Switzerland) at about 40 ^o^C. The dried and powder samples of each were weighed and packed in glass vials and stored in desiccators over silica gel until required for use. The percentage yield was calculated as:

$$ \mathrm{Percentage}\ \mathrm{yield} = \frac{\mathrm{Amount}\ \mathrm{of}\ \mathrm{extract}\ \mathrm{obtained}}{\mathrm{Amount}\ \mathrm{of}\ \mathrm{initial}\ \mathrm{sample}} \times 100\% $$

## Phytochemical screening

Aqueous and absolute methanol extracts of *V. sinaiticum* were screened for the presence of phytochemical following standard methods: alkaloids, phenolic compounds [27, 28], Anthraquinones [29], flavonoids [30], glycoside, saponins, tannins [31], Steroids, and terpenes [32].

## Study site selection and test organism

The selection of the study site was based on the information from the National Tsetse and Investigation and Control Centre (NTTICC), and the National Animal Health Diagnostic and Investigation Center (NAHDIC) located in Bedele town, and previous reports on the prevalence of animal trypanosomiasis in the area [20, 33–35]. The test organism, *T. congolense* was acquired from infected cattle at Sebategna kebele of Ilu-Aba-Bora-Zone, 480 km South West of Addis Ababa where the cattle in this area are known for drug resistance in cattle. However, the sensitivity of the test organism to existing drugs was not known. The blood sample was collected by NTTICC expert from the peripheral ear vein of each animal following the methodology in Mergia et al. [36]. The presence of *T. congolense* was detected by Microhaematocrit Buffy Coat Technique (MHBCT) [37] and Murray et al. [38]. Blood from the jugular vein of an animal with peak parasitaemia of (~10^8^ trypanosomes/ml) [39] was collected to the ethylene diamine tetra acetic acid (EDTA) coated tube and diluted with PBS. Then 0.2 ml of blood (~10^4^ trypanosomes/ml) was injected intraperitoneally to six laboratory mice and transported to Aklilu Lemma Institute of Pathobiology, Addis Ababa University for serial passage to other mice. About 0.2 ml of blood collected from the donor mice containing (~10^4^ trypanosomes/ml) [39] was injected intraperitoneally into mice that were acclimatized to laboratory conditions and were used for in vivo evaluation of the crude leaf extracts of *V. sinaiticum* [36].

## Experimental animals

Healthy Swiss albino mice (weighing 20–30 g and age of 8–12 weeks) were obtained from the Ethiopian Health and Nutrition Research Institute and School of Pharmacy, Addis Ababa University. Animals were housed in polypropylene cages (6–10 animals per cage), maintained under 12 hr light/dark cycle and allowed free access to a pellet diet and clean water *ad libitum*. All procedures comply with the guide for the care and use of laboratory animals [39–41].

## Determination of acute toxicity

The acute toxicity study was conducted in two phases, each with nine female Swiss albino mice. After seven days of adaptation, each extract, aqueous and methanol was dissolved in 2 % Tween 80 in sterile water and given through intraperitoneal route. In the first phase, three groups, each with three mice were given 10, 100, and 1000 mg/kg body weight of doses of each extract, respectively. In the second phase, three mice within each group were given 1600, 2900, and 5000 mg/kg doses of each extract, respectively to determine the appropriate lethal dose (LD_50_) value. Also, a third group of six mice (three mice for each phase) was set up as a control group and received the reconstituting solvent 2 % Tween 80 in sterile water. All animals were kept under strict observation for behavioral, neurological, autonomic or physical changes such as alertness, motor activity, restlessness, convulsions, coma, diarrhea and lacrimation for 24 h, with special attention during the first four hours. These observations continued for further 14 days for any signs of overt toxicity. Then the lowest dose, which killed one mouse (minimum toxic dose) and the highest dose, which had not killed any mouse (maximum tolerated dose) were noted, and the geometric mean of these two doses gave LD_50_. The LD_50_ was computed using the formula:

$$ {\mathrm{LD}}_{50} = \sqrt{\mathrm{Minimum}\ \mathrm{Toxic}\ \mathrm{Dose} \times \mathrm{Maximum}\ \mathrm{Tolerated}\ \mathrm{Dose}} $$

## Determination of in vivo antitrypanosomal activities

### Parasite inoculation and extracts administration

Forty healthy Swiss albino mice infected intraperitoneally with 0.2 ml of *T. congolense* infected blood (~10^4^trypanosomes/ml) were used to test trypanocidal activities of aqueous and methanol extracts. The mice were randomly divided into eight groups each comprising of 5 mice. The treatment of mice with the extracts in each group began on the 12th day of post-infection (day 0 of treatment) when the infected mice showed peak parasitaemia (~10^8^ trypanosomes/ml). Group I-III were given *V. sinaiticum* aqueous extract (VSAE) of 100, 200, 400 mg/kg; group IV-VI were given *V. sinaiticum* methanol extract (VSME) of 100, 200, 400 mg/kg, respectively. The positive control, group VII was treated with diminazine aceturate (Veriben®) (DA28) at a single dose of 28 mg/kg bwt while for the negative control, group VIII was given 2 % Tween 80 (TW80). On each day of drug administration, the aqueous and methanol extracts of *V. sinaiticum* were freshly prepared by solubilizing in 2 % Tween-80 in sterile water and administered intraperitoneally in the morning consecutively for seven days. The doses were selected based on the acute toxicity study. The middle dose was one tenth of the lethal dose (~2000 mg/kg), higher dose was twice the middle dose, and the lower dose was half of the middle dose [41].

### Determination of parasitaemia

A drop of blood obtained from a mouse by tail snipping was used to make smears on the slides and to monitor parasitaemia every other day microscopically at 400× total magnification. The degree of parasitaemia was determined using the “Rapid Matching” method of Herbert and Lumsden [38]. The wet smear was prepared in triplicates from each animal, and the mean value of slide counts was taken per sample examined microscopically. Logarithm values of these counts were obtained by matching with the table given by Herbert and Lumsden [38].

$$ \%\ \mathrm{change}\ \mathrm{in}\ \mathrm{parasitemia}\kern0.5em  = \frac{\mathrm{Mean}\ \mathrm{parasitemia}\ \mathrm{on}\kern0.5em \mathrm{DAY}\kern0.5em 14-\mathrm{Mean}\ \mathrm{Parasitemia}\ \mathrm{on}\ \mathrm{DAY}\kern0.5em 0}{\mathrm{Mean}\ \mathrm{Parasitemia}\ \mathrm{on}\ \mathrm{DAY}\kern0.5em 0} \times 100 $$

### Determination of packed cell volume (PCV)

Packed cell volume was measured using Wintrobe and Landsberg [42], and Wernery et al. [43] methods to predict the effectiveness of the test extracts in preventing hemolysis resulting from increasing parasitaemia associated with trypanosomiasis. It was monitored for infection three times until the 14th day (on day 0, 7, 14). 12 DPI was an important day to monitor since the treatment of mice with the extracts in each group began on the 12th day of post-infection (day 0 of treatment) when the infected mice showed peak parasitaemia (~10^8^ trypanosomes/ml). The effect of extracts in improving the PCV of treated animals was compared with the controls.

$$ \%\ \mathrm{change}\ \mathrm{in}\ \mathrm{P}\mathrm{C}\mathrm{V} = \frac{\mathrm{Mean}\ \mathrm{P}\mathrm{C}\mathrm{V}\ \mathrm{on}\ \mathrm{DAY}\kern0.5em 7-\mathrm{Mean}\ \mathrm{P}\mathrm{C}\mathrm{V}\ \mathrm{on}\ \mathrm{DAY}\kern0.5em 0}{\mathrm{Mean}\ \mathrm{P}\mathrm{C}\mathrm{V}\ \mathrm{on}\ \mathrm{DAY}\kern0.5em 0} \times 100\kern0.5em \mathrm{and}\ \mathrm{on}\ 14\mathrm{D}\mathrm{P}\mathrm{I}? $$

### Determination of body weight

The body weight (in gram) of each mouse in all groups was measured before infection, on the day treatment commenced (day 0) and every other day (on Day 2, Day 4, Day 6, Day 8, Day 10, Day 12 and Day 14) up to day 14.

$$ \%\ \mathrm{change}\ \mathrm{in}\ \mathrm{Body}\ \mathrm{weight} = \frac{\mathrm{Mean}\ \mathrm{body}\ \mathrm{weight}\ \mathrm{on}\ \mathrm{Day}\kern0.5em 14-\mathrm{Mean}\ \mathrm{Body}\ \mathrm{weight}\ \mathrm{Day}\kern0.5em 0\ }{\mathrm{Mean}\ \mathrm{Body}\ \mathrm{weight}\ \mathrm{on}\ \mathrm{Day}\kern0.5em 0} \times 100 $$

### Determination of mean survival time

Mortality was monitored daily and the number of days from the date of inoculation of the parasite to death was recorded for each mouse in the treatment and control groups throughout the follow-up period for six weeks. The Mean Survival Time (MST) for each group was calculated as follows;

$$ \mathrm{Mean}\ \mathrm{survival}\ \mathrm{time} = \frac{\mathrm{Sum}\ \mathrm{of}\ \mathrm{survival}\ \mathrm{time}\ \mathrm{of}\ \mathrm{a}\mathrm{ll}\ \mathrm{mice}\ \mathrm{in}\ \mathrm{a}\ \mathrm{group}\ \left(\mathrm{days}\right)}{\mathrm{Total}\ \mathrm{number}\ \mathrm{of}\ \mathrm{mice}\ \mathrm{in}\ \mathrm{that}\ \mathrm{group}} $$

## Data analysis

The data obtained from the study were summarized and expressed as mean ± standard error of mean (SEM). Data analysis was performed using Statistical Package for Social Science (SPSS), version 17.0. One-way ANOVA followed by Tukey’s multiple comparison tests were carried out to compare the results obtained from different groups and to determine statistical significance. P values less than 0.05 were considered significant.

## Results

### Yield for plant extract and phytochemical screening

The aqueous and methanol leaf extracts of *V. sinaiticum* gave 13.09%w/w and 18.13%w/w respectively. The phytochemical screening results are shown in Table 1. The methanol extract had more phytochemicals compared to the aqueous extract; however, anthraquinones and terpenes were absent in both extracts.

**Table 1:** Phytochemicals screened from the aqueous and methanol leaf extracts of *Verbascum sinaiticum*

Constituents	*V. sinaiticum*	
	Aqueous extract	Methanol extract
Alkaloids	+	+
Anthraquinones	-	-
Flavonoids	-	+
Glycoside	-	+
Saponins	+	-
Steroids	-	+
Phenolic compounds	-	+
Tannins	-	+
Terpenes	-	-

+ = present, - = absent

### Acute toxicity test

The acute toxicity bioassay showed that the Lethal Dosage (LD_50_) of the aqueous (LD_50_ = 3807.9 mg/kg) and methanol (LD_50_ = 2154.1 mg/kg) leaf extracts of *V. sinaiticum* was above 2000 mg/kg and there was no evidence of an acute toxicity at the doses tested indicating good safety margin.

## In vivo antitrypanosomal activity of aqueous and methanol crude extracts

### Effect on parasitaemia

The reduction of parasitaemia showed variation among the administered doses of aqueous and methanol extracts of *V. sinaiticum*. The animals treated with 400 mg/kg dose of the aqueous extract had significantly (*p* < 0.001) low level of parasitaemia on days 8 and 10 when compared with other doses of the aqueous extract treated mice. The methanol leaf extract of *V. sinaiticum* at 100 mg/kg, 200 mg/kg and 400 mg/kg had kept parasitaemia at a significantly low level on day 6, 8, 10, 12 and 14 (*p* < 0.001) as compared with the negative control (Table 2).

**Table 2:** The effect of aqueous and methanol leaf extracts of *Verbascum sinaiticum* on parasitaemia level of *Trypanosoma congolense* infected mice

Days	Parasitaemia level (log number/ml)							
	DA28	TW80	VSAE100	VSAE200	VSAE400	VSME100	VSME200	VSME400
Day0	7.68 + 0.18	7.44 + 0.17	7.32 + 0.07	7.08 + 0.07	7.31 + 0.11	7.44 + 0.11	7.08 + 0.15	7.32 + 0.12
Day2	0.00 + 0.00^d^***	7.74 + 0.17	7.56 + 0.11	7.44 + 0.11	7.56 + 0.11	7.68 + 0.12	7.56 + 0.11	7.62 + 0.12
Day4	0.00 + 0.00^d^***	7.86 + 0.11	7.50 + 0.09	7.80 + 0.13	7.32 + 0.07^a^**^,^^c^*	7.50 + 0.16	7.32 + 0.07^a^*	7.26 + 0.11^a^**
Day6	0.00 + 0.00^d^***	8.16 + 0.11 ^d^***	7.32 + 0.00^a^***	7.44 + 0.17^a^***	7.14 + 0.11^a^***	7.44 + 0.11^a^***	7.02 + 0.07^a^***^,^^b^*	6.96 + 0.11^a^***^,^^b^*
Day8	0.00 + 0.00^d^***	8.28 + 0.07^d^***	7.44 + 0.06^a^***	7.38 + 0.07^a^***	6.78 + 0.12^d^***	7.68 + 0.12^a^***	6.66 + 0.06^ab^***	6.54 + 0.11^ab^***
Day10	0.00 + 0.00^d^***	8.52 + 0.12 ^d^***	7.74 + 0.11^a^***	7.62 + 0.07^a^***	7.08 + 0.12^d^***	7.86 + 0.11^a^***	7.14 + 0.11^ab^***	6.36 + 0.17^abc^***
Day12	2.16 + 1.32^d^***	8.64 + 0.06	8.04 + 0.11	7.92 + 0.12	7.56 + 0.11	7.98 + 0.07	7.44 + 0.11	6.72 + 0.20
Day14	5.52 + 0.07^d^***	8.82 + 0.12	8.40 + 0.09	8.16 + 0.11^a^***	8.04 + 0.11^a^***	8.28 + 0.07^a^*	7.74 + 0.11^a^***^,^^b^*	7.20 + 0.16^ab^***^,^^c^*

Values are in Mean ± SEM; *N* = 5; *DA28* diminazine aceturate 28 mg/kg-the positive control; *VSAE V. sinaiticum* aqueous extract; ^a^compared to TW80 = 2 % tween 80-the negative control; ^b^compared to VSME 100 mg/kg; ^c^compared to VSAE 200 mg/kg; ^d^compared with all groups; *p < 0.05, **p < 0.01 and ***p < 0.001

### Effect on packed cell volume

**Fig. 1:**
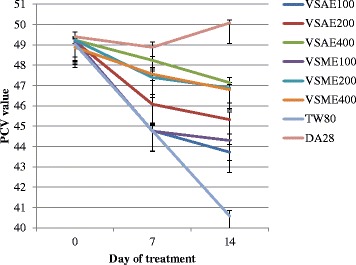
Comparison of the effect of aqueous and methanol leaf extracts of *Verbascum sinaiticum* on packed cell volume of *Trypanosoma congolense* infected mice

Animals treated with a higher dose (400 mg/kg) of the aqueous extract of *V. sinaiticum* had a statistically significant (*p* < 0.001) higher PCV value (47.14 ± 0.25) as compared to the negative control group (40.58 ± 0.28) on day 14 of treatment. Analysis of change in the percentage of PCV from day 7 to day 14 of treatment also showed that the aqueous extract at 200 and 400 mg/kg dose had prevented a drop in PCV associated with trypanosomes as compared to the negative control group (Table 3). In consistence with the results seen in PVC (Fig. 1), animals treated with the methanol extract of *V. sinaiticum* had higher PCV value (*p* < 0.001) as compared to the negative control groups at the end of the observation period (Table 3).

**Table 3:** Effect of the aqueous and methanol leaf extracts of *Verbascum sinaiticum* on packed cell volume of *Trypanosoma congolense* infected mice

Days	PCV Values							
	DA28	TW80	VSAE100	VSAE200	VSAE400	VSME100	VSME200	VSME400
Pre-infection	51.18 ± 0.37	50.88 ± 0.14	51.38 ± 0.18	51.18 ± 0.24	51.44 ± 0.12	51.22 ± 0.19	51.30 ± 0.27	51.48 ± 0.23
Day 0	49.40 ± 0.23	49.04 ± 0.29	49.10 ± 0.19	49.18 ± 0.18	49.24 ± 0.16	49.20 ± 0.22	49.24 ± 0.17	48.88 ± 0.20
Day 7	48.88 ± 0.25^d^**	44.78 ± 0.37	44.78 ± 0.36	46.08 ± 0.32^a^*^,^^b^*	48.24 ± 0.18^abc**^	44.76 ± 0.27	47.40 ± 0.41^ab^**	47.56 ± 0.16 ^ab^**
%change PCV day 7–0	−1.05	−8.69	−8.79	−6.3	−2.03	−9.02	−3.73	−2.7
Day 14	50.08 ± 0.15^d^**	40.58 ± 0.27^d^**	43.72 ± 0.39^a^**	45.32 ± 0.37^a**, b*^	47.14 ± 0.25^d^**	44.30 ± 0.32 ^a^**	46.80 ± 0.18 ^ab^**	46.88 ± 0.35 ^ab^**
% change PCV day 7–14	2.45	−9.38	−2.36	−1.65	−2.28	−1.02	−1.09	−1.59

Values are expressed in Mean ± S.E.M; *N* = 5; *DA28* diminazine aceturate 28 mg/kg- the positive control; *VSAE V. sinaiticum* aqueous extract; ^a^compared to TW80 = 2 % tween 80-the negative control; ^b^compared to VSAE 100 mg/kg; ^c^compared to VSAE 200 mg/kg; ^d^compared with all groups; *p < 0.05 and **p < 0.001

### Effect on body weight

**Fig. 2:**
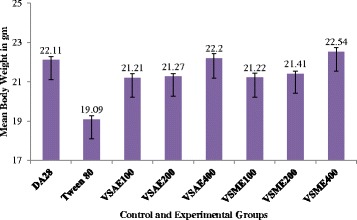
Comparison of the effect of aqueous and methanol leaf extracts of *Verbascum sinaiticum* on body weight of *Trypanosoma congolense* infected mice

The aqueous and methanol extracts of *V. sinaiticum* were capable of improving body weight of treated animals on days 8–14 as compared to the negative control group (*p* < 0.001). Animals treated with 400 mg/kg dose of both extracts of *V. sinaiticum* had a significantly (*p* < 0.001) higher body weight as compared to the negative control group (19.09 ± 0.34) on day 14 of treatment (Fig. 2).

### Effect on mean survival time

**Fig. 3:**
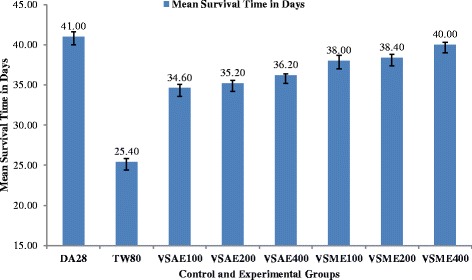
Mean survival time of *Trypanosoma congolense* infected mice treated with aqueous and methanol crude leaf extracts of *Verbascum sinaiticum*

Animals treated with 400 mg/kg of the methanol extract of *V. sinaiticum* had the highest mean survival time (40.20 ± 0.31 days) as compared to the negative control group (25.40 ± 0.43) while animals that received the positive control diminazine aceturate had a mean survival time of 44.00 ± 0.63 days (Fig. 3).

**Fig. 4:**
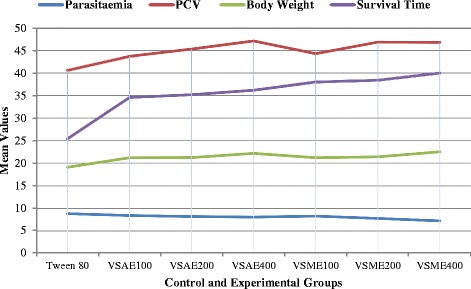
Comparison of the effects of aqueous and methanol leaf extracts of *Verbascum sinaiticum* on parasitaemia, packed cell volume, body weight, and survival time of *Trypanosoma congolense* infected mice at the end of the experimental study

The overall activities of the extracts are shown in Fig. 4. The extracts had shown an increased values in the indices used for studying the potential of *Verbascum sinaiticum* against *Trypanosoma congolense* infected mice. The PVC and survival time had higher values as compared to negative control.

## Discussion

The *Verbascum sinaiticum* extracts showed an LD_50_ above 2000 mg/kg indicating there was no lethal effect. The results validate why the plant has been used by several traditional healers for treatment of various disease including animal trypanosomiasis [21–24, 26], and toxicity and antidotes have not been reported. The experimental determination of this safety margin would justify that the plant is safe at the dose levels used in this study, which is an additional proof of the medicinal value of the plant in folk medicine. Though in previous studies, phytochemicals flavonolignans, hydrocarpin, sinaiticin, as well as two flavones, chrysoeriol and luteolin from the leaf of *V. sinaiticum* exhibited dose-dependent cytotoxicity when tested against cultured P-388 cells [44].

The trypanocidal and trypanostatic efficacy of *V. sinaiticum* aqueous and methanol extracts are associated with the presence of one or more biological active principals. This was shown to be true by the positive test for the presence of alkaloids, flavonoids, phenols, glycosides, saponins, steroids, and tannins. Tatli et al. [45] reported that *Verbascum* L. species has considerable saponins, iridoid and phenylethanoid glycosides, monoterpene glucoside, neolignan glucosides, flavonoids, steroids and spermine alkaloids that are responsible for biological activities thus their use in folk medicines. Previous in vitro and in vivo studies conducted on the antitrypanosomal activities of these phytochemicals have reported the trypanocidal and trypanostatic potential of these compound in human and animal trypanosomes [14, 15, 46–48]. Therefore, the observed in vivo antitrypanosomal activity of *V. sinaiticum* might be attributed either to the individual class of compounds antitrypanosomal activity or to the synergistic effect of each class of compounds in the extracts [49].

The reduction of parasitaemia (6.36 + 0.17) and prolonging of the lifespan (40.20 ± 0.31 days) of infected mice by a higher dose of methanol extract can be associated to the trypanosuppression of the phytochemical mainly the flavonoids that have been shown to exhibit potential to inhibit the growth of African Trypanosomes [14, 50]. These biologically active phytochemicals act at a single or multiple target sites associated with a physiological process [14] and interference with the redox balance of the parasites acting on the respiratory chain or on the cellular defenses against oxidative stress that partially eliminates the trypanosomes [51, 52]. Moreover, the flavonoids compounds have demonstrated promising antitrypanosomal activities on the trypomastigote forms, which are usually found in the bloodstream of mammalians [53, 54]. Alkaloids, flavonoids, phenolics, and terpenes have shown trypanocidal activity in an in vitro investigation, and the alkaloids have been shown to reduce the growth of trypanosomes by intercalating in the deoxyribonucleic acid (DNA) of trypanosomes [13] and inhibiting protein synthesis [53].

The trypanosomes are not eliminated from the blood stream of infected mice though animals treated with 400 mg/kg methanol extract had significantly (*p* < 0.001) lower mean parasitaemia (7.20 + 0.16) as compared to the negative control group (8.82 + 0.12) on day 14 of treatment. This result is similar to other studies conducted on *Khaya senegalensis* [55], *Artemisia abyssinica* [56], *Adansonia digitata* [18], *Garcinia kola* [57] and *Carrisa edulis* [58] with antitrypanosomal activities. The efficacy of crude extract might be masked with high parasite load in the host animal [55, 59], or it could be due to enzymatic inactivation of active compounds of the phytochemical in the host animal and impaired absorption from the site of administration [52, 60]. In addition, the concentration of the phytochemicals that reaches the target organs, the duration the phytochemicals take to effect a cure, and short half-life of the phytochemicals can reduce the efficacy of the crude extracts [61].

The effectiveness of diminazine aceturate was challenged in all mice approximately on days 12–14 of treatment and relapse of parasitaemia was observed [18]. *T. congolense* sequester in small vessels and capillaries of the heart, skeletal and other tissues, which often leads to a prolonged pre-patent period [62]. The relapse is also a clue to the existence of drug resistance trypanosomes in the South West and North West part of the country since the samples were acquired from the south-west part of Ethiopia. The test organism obtained from south-west part of Ethiopia can be a heterogeneous population of trypanosomes that are sensitive and resistant to diminazine aceturate; hence, the treatment with diminazine aceturate could have eliminated the sensitive sub-population through its therapeutic effects, so that the relapse is the manifestation of resistant population [6, 7, 63].

The reduction in packed cell volume (PCV) observed in extracts treated mice compared to diminazine aceturate treated mice could be due to acute hemolysis induced by the growing infection and increased susceptibility of red blood cell membrane to oxidative damage [64]. A higher PCV exhibited in mice treated with the extracts compared with the negative control group might be as a result of the phytochemicals reducing the parasite load, neutralizing the toxic metabolites produced by trypanosomes; the aetiological factors involved in the haemolysis of red blood cells and scavenging the trypanosome associated free radicals [59, 65, 66]. It could also be attributed to the potential antioxidant activity of the flavonoids and glycosides present in the leaf, which was also confirmed by similar studies previously done on related plant species from the same family [67–69].

The loss of body weight is associated with progression of infection followed by appetite decreases, and the animal loses condition as a result, there is wasting. The decreased supply of oxygen because of the anemia is also an important factor [70, 71]. However, animals, which received 400 mg/kg aqueous and methanol extract of *V. sinaiticum* gained weight by 1.07 and 1.67 % respectively, which was statistically significant (*p* < 0.001) as compared to the negative control groups. This shows that because of reduction in parasitaemia and prevention of drop in PCV as a result of the trypanosuppressive effect of the extracts against trypanosome infection, physical status of the treated mice was improved. Similar observations have been made by other researchers [59, 68, 69, 72–74].

## Conclusion

In vivo tests usually show reliable antitrypanosomal activities of traditional antitrypanosomal medicinal plants. *V. sinaiticum* trypanocidal activity analysis indicated its antitrypanosomal potential and without toxicity effect on the host organism. The phytochemicals identified are known to have antitrypanosomal activities. The crude extracts have partially eliminated trypanosomes in a dose-dependent manner. Though unintentional, this study has also shown the existence of drug-resistant trypanosomes in the field stock. The study has shown that *V. sinaiticum* has a potential to be used as trypanocidal though further analysis is required to identify potent biologically active chemicals.

## Abbreviations

AAT: African Animal Trypanosomiasis
ANOVA: Analysis of Variance
bwt: Body weight
DA28: Diminazine aceturate (Veriben®)
DNA: Deoxyribonucleic acid
DPI: Days Post-Infection
EDTA: Ethylene diamine tetra acetic acid
LD_50_: Lethal dose
MHBCT: Microhaematocrit Buffy Coat Technique
MST: Mean survival time
NAHDIC: National Animal Health Diagnostic and Investigation Center
NTTICC: National Tsetse and Trypanosomosis Investigation and Control Centre
PCV: Packed cell volume
SEM: Standard error of mean
SPSS: Statistical Package for Social Science
TW80: Tween 80
VSAE: *V. sinaiticum* aqueous extract
VSME: *V. sinaiticum* methanol extract
